# Neuroprotection by Drugs, Nutraceuticals and Physical Activity

**DOI:** 10.3390/ijms24043176

**Published:** 2023-02-06

**Authors:** Andrea Tarozzi, Cristina Angeloni

**Affiliations:** Department for Life Quality Studies, Alma Mater Studiorum, University of Bologna, Corso d’Augusto 237, 47921 Rimini, Italy

Acute and chronic neural injuries, including stroke, brain trauma and neurodegenerative diseases such as amyotrophic lateral sclerosis (ALS), Huntington’s disease (HD), Parkinson’s disease (PD), and Alzheimer’s disease (AD) are associated with high morbidity and mortality rates. The symptoms and exacerbations of these diseases are, however, very different according to their specific pathways of neuronal impairment. Several mechanisms can lead to neurodegeneration, including calcium overload, excitatory amino acid release, oxidative stress, inflammation and microglial activation, protein misfolding, proteostasis and mitochondrial dysfunction [1]. The clinical management of these diseases is currently very critical, with therapeutic strategies often limited to relieving symptoms rather than treating the disease. The scientific community, in addition to focusing on the development of effective new neuroprotective drugs, is also exploring non-pharmacological approaches relying on food components, such as nutraceuticals, and physical activity. In this regard, several studies have shown that nutraceuticals and physical activity have similar or complementary neuroprotective mechanisms, suggesting the need to integrate new approaches with the pharmacological interventions in order to enhance neuroprotective effects.

This Special Issue focuses on the effect of drugs, nutraceuticals, and physical activity, whether alone or in combination, necessary to counteract neurological disorders and contains twenty-four contributions: twenty research articles, three reviews, and one brief report addressing the most recent advances in this area.

Defining the pathogenetic mechanisms of neurodegenerative diseases and neurological disorders is a fundamental requirement for identifying new pharmacological targets and neuroprotective treatments.

In this regard, Lillo et al. [2] evaluated the expression and function of the A2A/A3 adenosine receptor heteromer (A2AA3Het) in neuronal and microglial primary cultures. The A2AA3Het expression was higher in striatal than in cortical and hippocampal neurons, whereas it was similar in resting and activated microglia. Further, the assessment of the A2AR or A3R function confirmed that signaling via these receptors depends on the context, that is, on the structure of the heteromer and/or allosteric interactions with G and scaffold proteins. A2AA3Het was upregulated in microglia from the APPSw, Ind model of AD. In the same model, the functional selectivity was such that A2AR increased Gi-mediated signaling of A3R, suggesting that A2AR antagonists deserve consideration in the treatment of AD.

Jordan et al. [3] focused on the role of Rab GTPases, small proteins that play crucial roles in vesicle transport and membrane trafficking, in neurodegeneration. The reviewed studies provided strong evidence that Rab GTPases are not only heavily involved in AD pathology, but also that modulation of Rab expression and activity could rescue associated defects and toxicity. These studies also offered the potential for a multifaceted approach to treatment, incorporating different Rabs to target multiple aspects of AD pathology at once.

Growing attention is being paid to the biological effects of microwave on brain functions. Zhi et al. [4] explored the regulatory mechanisms of synapsin I (SYN1) fluctuations following microwave exposure and its subsequent effect on GABA release and cognitive function. The rats exposed to microwaves showed abnormality in synapse formation and neuronal structure as well as behavioral and cognitive dysfunction and GABA transmission abnormalities. In particular, they showed that abnormal decreases in the p-SYN1 can lead to obstacles in the vesicular anchoring of GABA, a process induced by the increase in Cdk5 activated via the Calpain-p25 pathway following microwave exposure. Although p-Erk may play a role in cognitive impairment after microwave exposure, further investigation of its specific function in this process is still needed.

Hassan et al. [5] evaluated a potential link between mitochondrial dysregulation and idiopathic autism spectrum disorder (ASD) by focusing on mitochondrial respiratory capacity and membrane potential in lymphoblastoid cell lines (LCL), derived in turn from an autistic child (ALCL) and a non-autistic sibling (LCL) using high-resolution respirometry. The respiratory capacities of oxidative phosphorylation, electron transfer of the Complex I and Complex II-linked pathways, and Complex IV activity of the ALCL were significantly higher compared to the LCL control. Mitochondrial membrane potential of ALCL was also significantly higher than LCL, linking the mitochondrial dysfunction with ASD.

Paulo et al. [6] focused on the relationships between high caloric diet-induced obesity and cognitive impairment in aged rats. Male rats were kept on a high caloric intake diet for 2 years. During this period, rats developed obesity and displayed significant long-term recognition memory impairment compared to age-matched controls. These obese rats evidenced a reduction in the magnitude of long-term potentiation in the hippocampus, associated with a reduction in the levels of the brain-derived neurotrophic factor receptors TrkB full-length. The results of this study underline that chronic high caloric diets can worsen age-associated cognitive decline, a process which is likely related to impaired synaptic plasticity, an issue associated with deficits in TrkB-FL signaling.

The flavoprotein kynurenine 3-monooxygenase (KMO) is localized in mitochondria and catalyzes the synthesis of 3-hydroxykynurenine, a key step in the kynurenine pathway (KP) of tryptophan degradation. The impairment of KP metabolism due to inflammation has long been associated with the pathogenesis of several neurodegenerative disorders, including HD, which is caused by the expansion of a polyglutamine stretch in the huntingtin (HTT) protein. Swaih et al. [7] showed that expansion of the disease-causing polyglutamine tract in HTT leads to the formation of proteinaceous intracellular inclusions that disrupt this interaction with KMO. They also assessed KMO and HTT localization within the cell, finding that the KMO-HTT interaction is localized to the outer mitochondrial membrane. These data suggest that KMO may interact with a pool of HTT at the mitochondrial membrane, highlighting a possible physiological role for mitochondrial HTT. In addition, KMO-HTT interaction was found to be abrogated upon polyglutamine expansion, indicating a heretofore unrecognized relevance in the pathogenesis of HD.

Several studies have established that a reduction in the levels of the cellular prion protein (PrPC) is a promising avenue for the treatment of prion diseases. Eid et al. [8] identified a cardiac glycoside (CG) with characteristics which are more favorable to lowering steady-state PrPC levels in the brain for the treatment of prion diseases. In this context, the authors focused on C40-dehydro-oleandrin as a CG derivative that reaches higher levels in the brain than in the heart after subcutaneous administration. C40-dehydro-oleandrin also exhibited promising pharmacological properties, and suppressed steady-state PrPC levels in cells of human neuronal and astrocytic lineage through a mechanism of action that requires the direct engagement of C40-dehydro-oleandrin with its Na,K-ATPases target.

Epilepsy is one of the most common neurological chronic disorders characterized by repeated seizures [9]. Hyder Pottoo et al. [10] investigated a combination of sodium valproate, baclofen and thymoquinone for both antiseizure and neuroprotective potential by using electrical stimulation of neurons to induce maximal electroshock (MES) in rats. They demonstrated the synergistic effects of this drug combination against MES-induced seizures through its ability to reduce neuronal death, inflammatory markers (IL-1β, IL-6) and mTOR at hippocampus level. They also confirmed the neuroprotective effects of drug combination in an in vitro seizure model using pentylenetetrazol and HEK-293 cells.

Furuta et al. [11] studied the neuroprotective mechanisms of melatonin via mitochondrial melatonin receptors (MTs) during the ischemic postconditioning (PostC) phenomenon in hippocampal CA1 pyramidal cells extracted from C57BL mice. Melatonin and an MT agonist, ramelteon, suppressed the NMDAR currents and potentiated the depolarization of mitochondrial membrane potentials after ischemic challenge. These findings were confirmed using luzindole and cyclosporine A, an MT antagonist and mitochondrial permeability transition pore inhibitor, respectively. In particular, the results demonstrate that melatonin-induced PostC inhibits the influx of Ca^2+^ into cytoplasm by decreasing the NMDAR activity mediated through MTs.

Turovsky et al. [12] evaluated the neuroprotective effects of D4-Linoleic acid (D4-Lnn), which contains four deuterium atoms, against cell death induced by ischemia-like conditions (oxygen-glucose deprivation/reoxygenation, OGD/R) in a culture of mouse cortical neurons and astrocytes. D4-Lnn showed the ability to inhibit necrotic and apoptotic death in cortical neurons through several mechanisms such as the modulation of astrocyte reactivation and the regulation of several genes involved in the induction of cell death, inflammation, and excitotoxicity.

Peinado et al. [13] biosynthesized complex protein neuroglobin-hyaluronate nanoparticles (Ngb-NPs) to overcome the difficulty of experienced by Ngb in crossing the blood–brain barrier (BBB) and exerting neuroprotective effects at a brain level in an animal model of stroke (MCAO). They demonstrated that Ngb-NPs, when they are systemically, injected can cross the BBB and increase the survival of MCAO animals. Although no changes were found with the Ngb-NPs treatment, either in the infarcted brain volume or in the oxidative and nitrosative stress assessments, the proteomic approaches indicated that in identical treatment conditions Ngb-NPs changed the expression of proteins involved in some biological and regenerative processes, such as mitochondrial function, cell death, endocytosis, protein metabolism, cytoskeletal remodelling and synaptic function. Among the proteins modulated by Ngb-NPs, FBXO7, NTRK2 and ATX2L are the most relevant in the field of brain infarction.

Lee et al. [14] mainly reviewed the pharmacological treatments of Rho-associated kinase (ROCK) and phosphodiesterase-5 (PDE5) that affect dementia, such as AD or vascular dementia, in animal and in vitro studies. The authors especially focused on the pharmacological activities of ROCK and PDE-5 inhibitors to improve the different symptoms of dementia. In this regard, they also performed a meta-analysis of the results obtained in animal studies, affirming that both ROCK inhibitors and PDE-5 inhibitors mainly improved the cognitive impairment.

Spinocerebellar ataxia (SCA) is a rare genetic neurodegenerative disease that has no definitive cure [15]. Lin et al. [16] investigated the neuroprotective effects of insulin-like growth factor 1 (IGF-1), a downstream mediator of growth hormone, in SCA-type (SCA3) 84Q transgenic mice. The SCA3 84Q mice, treated with IGF-1 for 9 months, improved motor functions, enhanced the function of mitochondria, and reduced the degeneration of Purkinje cells as well as the oxidative stress and the ataxin-3 mutant protein levels in the cerebellum. The authors also highlighted that IGF-1 was not found to be carcinogenic in the SCA3 84Q mice at the dose and time used in this study, suggesting its therapeutic potential for treatment of SCA.

Neurological disorders are characterized by a multifactorial nature that requires treatment with molecules capable of targeting multiple pathogenic events. From this point of view, nutraceuticals could represent an effective therapeutic strategy thanks to their pleiotropic actions. In this Topic, several authors investigated the effect of one or a combination of different nutraceuticals in preventing or counteracting neurodegeneration. 

Abu-Elfotuh et al. [17] focused on the potential protective effects of sesamol, thymol, CoQ10, and wheat grass, either individually or in combination in a rat model of neurologic syndrome resembling PD induced by Manganese (Mn) overexposure. Sesamol and thymol were more effective in improving behavioral impairments induced by Mn compared to treatment with CoQ10 and wheat grass. Interestingly, the co-administration of the four nutraceuticals showed more pronounced improvements, which were confirmed by biochemical, molecular, and histopathological analyses. Moreover, the results suggest that sesamol and thymol neuroprotection is due, at least in part, to an interplay among Nrf2/HO-1, TLR4/NLRP3/NF-κB, GSK-3β and Bax/Bcl2 pathways.

Jeong et al. [18] studied the effect of an *Arecae pericarpium* ethanol extract against oxidative stress induced by glutamate in HT22 neuronal cells. The results showed that the *Arecae pericarpium* extract counteracted the cell death triggered by glutamate, reducing both intracellular reactive oxygen species and apoptosis. This extract increased the endogenous antioxidant defense system upregulating catalase, glutamate-cysteine ligase catalytic subunits, NAD(P)H quinone oxidoreductase 1, and heme oxygenase. This upregulation was attributed to the activation of phosphoinositide 3-kinase/protein kinase B and the translocation of Nrf2 to the nucleus.

Different phytochemicals from cannabis sativa demonstrated promising neuroprotective activities, reducing amyloid plaque deposition, stimulating hippocampal neurogenesis, and counteracting oxidative stress and neuroinflammation. On the bases of these recognized abilities, di Paolo et al. [19] studied the effects of cannabidiolic acid (CBDA), N-trans-caffeoyltyramine, and cannabisin B, obtained by hemp seeds, on the miRNome of neuron-like SH-SY5Y cells, focusing on the expression of miRNAs related to AD. They demonstrated that 31 microRNAs were modulated by these phytochemicals, of which miR-708-5p, miR-181a-5p, miR-190a-5p, miR-199a-5p, and miR-143-3p are specifically associated with AD. Overall, these data suggest that hemp seed metabolites could be considered as potential nutraceuticals to prevent/counteract neurodegeneration. 

Kang et al. [20] investigated the effect of a *Perilla frutescens* leaf extract (PFL) against vascular dementia in rats. This disease is characterized by a time-dependent memory deficit and neuroinflammation. PFL was given orally for 23 days. The rats, in which vascular dementia was induced, showed memory deficits, hippocampal neuronal death, and microglial activation. All these parameters were attenuated in rats treated with PFL. To clarify the molecular mechanisms behind the anti-inflammatory activity of the extract, a cell model of neuroinflammation was used. Microglial BV-2 cells were treated with PFL and activated by LPS exposure. PFL was able to reduce the production of NO, TNF-α, and IL-6 by modulating their upstream MAP kinases. These results suggest that PFL effect is, at least in part, associated with an attenuation of neuroinflammation.

Park et al. [21] explored the possibility of counteracting Charcot–Marie–Tooth disease (CMT) with farnesol, an acyclic sesquiterpene isoprenol. They focused on the ability of farnesol to improve the demyelinating phenotype in a mouse model of CMT type 1A. CMT is characterized by either the demyelination of Schwann cells or the degeneration of the peripheral axon. The in vitro results showed that farnesol facilitated myelin gene expression and ameliorated the myelination defect, while in vivo farnesol improved the peripheral neuropathic phenotype. Farnesol was able to increase motor nerve conduction velocity, compound muscle action potential, and the number and diameter of myelinated axons in CMT type 1A mice. Myelin protein zero was upregulated, while neural cell adhesion molecule, a demyelination marker, was downregulated by farnesol. These data suggest that farnesol administration could be a potential therapeutic strategy for the demyelinating type of CMT. 

Thymoquinone, the primary active substance found in *Nigella sativa* seeds, possesses several bioactivities with minimal chances of toxicity or side effects. Thanks to its low molecular weight and lipophilicity, it easily crosses the blood–brain barrier, being a potential substance for targeting the central nervous system. In their review, Pottoo et al. [22] explored the potential of thymoquinone for use in the treatment of neurological diseases. They evidenced the ability of thymoquinone to counteract oxidative stress and neuroinflammation, and also to reduce glutamate-induced apoptosis by suppressing mitochondrial dysfunction. It was also demonstrated that thymoquinone prevents the loss of dopaminergic neurons in various animal models of neurodegeneration. Finally, some clinical studies showed that thymoquinone can inhibit acetylcholinesterase activity. 

El-Nashar [23] et al. studied the effect of both an artichoke bract extract and solid lipid nanoparticles (SLNs) when loaded with this extract in streptozotocin-induced sporadic AD mice. The extract was characterized, and different phytochemical were identified, such as mono- and di-caffeoylquinic acids, apigenin, luteolin, and kaempferol O-glycosides. The extracts showed a high antioxidant activity in vitro. The treatments led to an improvement in cognitive functions and spatial memory recovery, accompanied by a reduction of inflammation and tau protein levels. Artichoke-loaded SLNs were more effective compared to respect to the free artichoke extract in protecting dentate Gyrus sub-regions. The results clarify the strong potential of artichoke bracts extract and suggest their potential use in therapeutic strategies to treat AD.

Over the last 15 years, accumulating evidence has shown that gut microbiota can alter brain pathophysiology by forming a bidirectional connection, commonly referred as the microbiota–gut–brain axis. Because of this, the relevance of an appropriate microbiota balance has been highlighted. Armeli et al. [24] investigated the anti-inflammatory effects of Milmed, a yeast extract obtained by treating Saccharomyces cerevisiae with electromagnetic waves, in murine microglial cells activated by LPS. Milmed yeast induces a reversal of LPS-M1 polarized microglia towards an anti-inflammatory M2-phenotype. In particular, Milmed treatment reduced the expression of the pro-inflammatory agents and increased the level of cell markers of the M2 anti-inflammatory phenotype. 

Physical exercise has been associated with a beneficial effect in neurodegenerative diseases because it improves the production of neurotrophic factors, neurotransmitters, and hormones. Moreover, exercise counteracts neuronal death and promotes neuroplasticity [25]. For these reasons and more, many studies have investigated the possibility of counteracting neurodegeneration with physical exercise, whether alone or in combination with drugs or nutraceuticals.

Based on these premises, Hamdan et al. [26] carried out a study on the neuroprotective activity of some phytochemicals such as morin, thymol, and thymoquinone in the absence/presence of physical and mental activities in a rat model of AD induced by aluminum chloride (AlCl_3_). AlCl_3_ led to a decline in spatial learning and memory, inducing histopathological changes in rat brains. The phytochemicals, in combination with physical and mental activities, counteracted oxidative stress by increasing the endogenous antioxidant system. Additionally, they blocked inflammasome activation, apoptosis, TLR4 expression and abnormal protein aggregation. Moreover, phytochemicals used in association with physical and mental activities reduced ApoE4 and LRP1 levels and regulated the Wnt3/β-catenin/GSK3β signaling pathway. The results of this study strongly suggest that the use of phytochemicals with physical and mental activities is a promising strategy for reducing AD. 

Although a positive role of physical activity in counteracting neurodegeneration has been widely demonstrated, the molecular mechanisms underlying this effect have not yet been fully clarified. With the aim to fill this gap, Santiago et al. [27] tried to dissect the molecular mechanisms behind the positive effect of physical activity in brain health. The results evidenced that the transcriptional patterns associated with physical activity significantly overlapped and negatively correlated with those of neurodegenerative diseases. Functional analysis indicated that physical activity could be neuroprotective in AD, PD, and HD by upregulating the synaptic signaling pathways. In other diseases such as frontotemporal dementia, the effects of physical activity are associated with the counteraction of mitochondrial dysfunction. This study identifies and confirms many molecular mechanisms by which physical activity exerts neuroprotection, evidencing both the unique and shared mechanisms involved. 

Gendi et al. [28] investigated the molecular mechanisms behind physical exercise and neurodegeneration using a rat animal model of hypoxic ischemic encephalopathy (HIE). The study was designed by considering four groups of rats: a sham sedentary group, a sham exercise training group, an HIE sedentary group, and an HIE exercise training group. The results showed that physical exercise improved motor and cognitive deficits and reduced the expression of proteins associated with apoptosis that were evidenced in HIE animals. The authors underlined that physical exercise induces neuroprotection through the stabilization of the mitochondrial cristae and the suppression of the apoptotic cascade.

In conclusion, the papers published in this Topic, both those addressing different levels of preventive/counteractive strategies and those investigating some neurodegenerative mechanisms as potential targets for neuroprotection, can be considered important contributions in the fight against these devastating pathologies.
